# Supplementary figures and images for: Meeting radiation dosimetry capacity requirements of population-scale exposures by geostatistical sampling
Source: PLoS One. 2020 Apr 24;15(4):e0232008. doi: 10.1371/journal.pone.0232008 (PMC7182271; doi:10.1371/journal.pone.0232008)

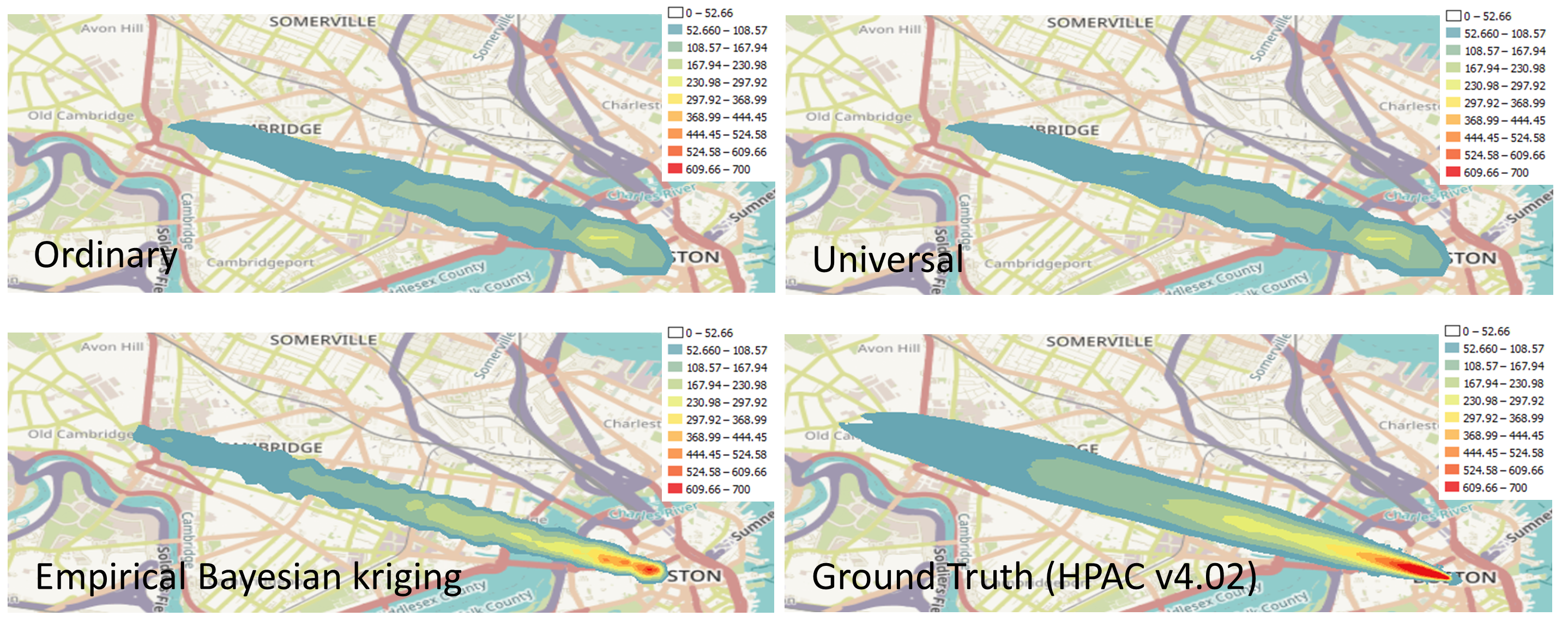

Supplement: S1 Fig — These plumes were generated with random points representing 1% of the Boston (N = 617,594) and Cambridge (N = 105,162) populations, the two subdivisions overlapping the Boston HPAC plume (no precipitation). These points were assigned dose values based on their location relative to the HPAC plume (N = 223 with dose > 0Gy). The following kriging methods were then applied to these data: Ordinary, Simple, Universal and Empirical Bayesian kriging (EBK). A contiguous plume could not be derived from Simple kriging. In these, and in other similar tests, the plume generated using EBK best resembled the plume produced by HPAC. (TIF) [file pone.0232008.s001.tif]

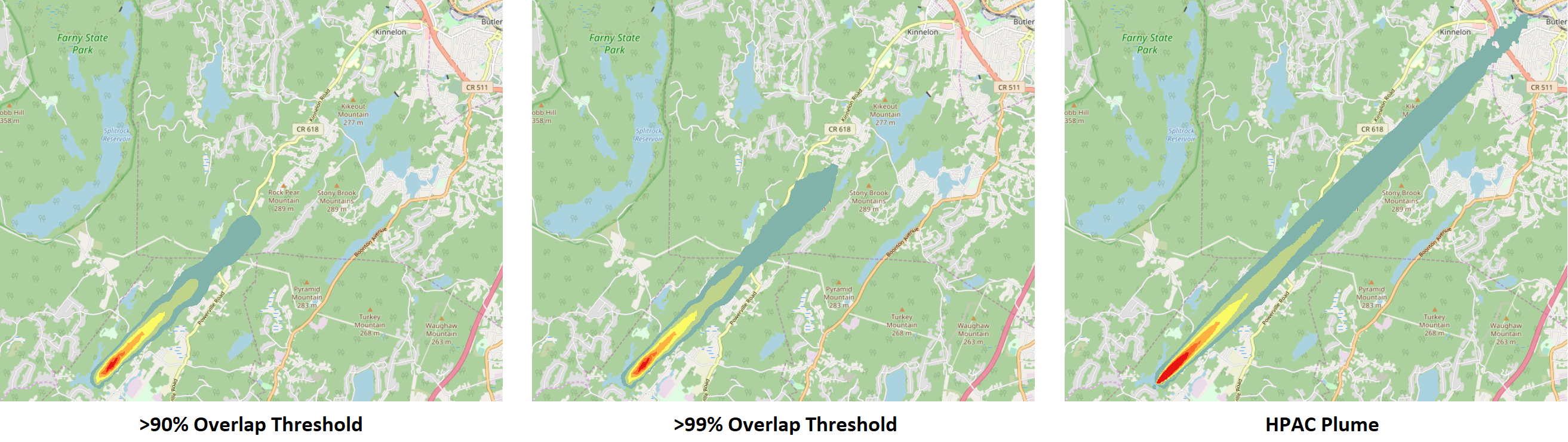

Supplement: S2 Fig — These plumes represent the radiation levels of the rural New York nuclear incident scenario. The first two plumes were derived using the geostatistical method using two iteration stopping criteria: a 90% (left plume) and a 99% (middle plume) stringency of overlap threshold between consecutive iterations. When compared to the HPAC plume (right plume), we found that increasing the stringency of overlap resulted in an additional 73 sampling locations selected by densification, which consequently significantly increased the size and similarity of the derived plume, most notably at the 1Gy and 2Gy contours. (TIF) [file pone.0232008.s002.tif]

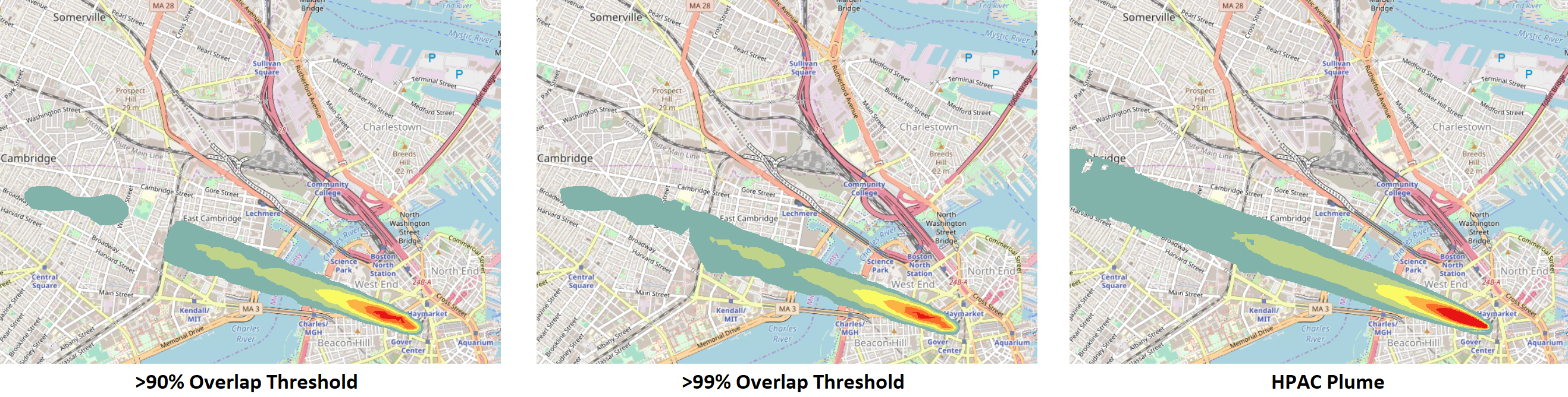

Supplement: S3 Fig — These plumes represent the radiation levels of the Boston MA nuclear incident scenario. The first two plumes were derived using the geostatistical method using two different stopping criteria: a 90% (left plume) and a 99% (middle plume) stringency of overlap between consecutive iterations. Right-most plume is HPAC. We find that the increased sampling of this scenario (43 additional sampling locations) led to the development of a gap in the 2Gy threshold, which resulted in a slight decrease in plume accuracy at the 2Gy threshold. (TIF) [file pone.0232008.s003.tif]
