# Supplementary material for: Meeting radiation dosimetry capacity requirements of population-scale exposures by geostatistical sampling
Source: PLoS One. 2020 Apr 24;15(4):e0232008. doi: 10.1371/journal.pone.0232008 (PMC7182271; doi:10.1371/journal.pone.0232008)
Supplement: S3 Table — (DOCX) [file pone.0232008.s006.docx]

**S2 Table. Simulated analyses of nuclear radiation with dose measurement error**

| City | Replicate | Original Simulation | | | Measurement Error Simul. (± 0.5 Gy) | | | Measurement Error Simul. (± 1.0 Gy) | | |
| --- | --- | --- | --- | --- | --- | --- | --- | --- | --- | --- |
|  |  | # of Iterations | # of Samples (>0Gy) | % accuracy of  ≥ 2Gy regions by population | # of Iterations | # of Samples (>0Gy) | % accuracy of  ≥ 2Gy regions by population | # of Iterations | # of Samples(>0Gy) | % accuracy of  ≥ 2Gy regions by population |
| Birmingham, AL | 2 | 4 | 167 | 69.6 | 4 | 195 | 61.7 | 5 | 160 | 56.6 |
| Boston, MA | 1 | 5 | 270 | 67.7 | 5 | 245 | 63.0 | 7 | 781 | 52.3 |
| Chicago, IL | 1 | 4 | 73 | 78.7 | 4 | 147 | 79.8 | 5 | 213 | 73.7 |
| Columbia, SC | 1 | 5 | 168 | 66.0 | 4 | 171 | 20.6 | 6 | 123 | 0.0* |
| Columbus, OH | 1 | 4 | 128 | 66.5 | 6 | 217 | 64.6 | 5 | 232 | 71.0 |
|  | 2 | 4 | 168 | 44.1 | 8 | 314 | 60.6 | 5 | 182 | 58.8 |
| * Columbia SC variance simulation did not lead to a plume with regions ≥2Gy due to failure of sampling (initial random points and densification-selected points) to overlap HPAC regions of high radiation. | | | | | | | | | | |
