# Supplementary material for: Meeting radiation dosimetry capacity requirements of population-scale exposures by geostatistical sampling
Source: PLoS One. 2020 Apr 24;15(4):e0232008. doi: 10.1371/journal.pone.0232008 (PMC7182271; doi:10.1371/journal.pone.0232008)
Supplement: S4 Table — (DOCX) [file pone.0232008.s007.docx]

**S3 Table: Samples Required for Plume Convergence is Inversely Related to Population Density**

| Scenario  (No Precipitation) | Average No. of Samples Required for Convergence^1^ | Standard Deviation  (Stdev) | Coefficient of Variation (Avg / Stdev) | Average Density of Region(s) Overlapping HPAC Plume^2^ |
| --- | --- | --- | --- | --- |
| Albany, NY | 183.7 | 45.2 | 24.6% | 4711 |
| Alexandria, VA | 180.7 | 24.8 | 13.7% | 9542 |
| Baltimore, MD | 162.0 | 12.2 | 7.5% | 7574 |
| Birmingham, AL | 154.7 | 21.4 | 13.8% | 1465 |
| Boston, MA | 211.3 | 52.0 | 24.6% | 16099 |
| Buffalo, NY | 189.3 | 69.3 | 36.6% | 6442 |
| Burlington, VT | 283.3 | 39.3 | 13.9% | 2710.5 |
| Camden, NJ | 304.3 | 90.4 | 29.7% | 8517.8 |
| Charleston, SC | 107.3 | 16.6 | 15.5% | 4434 |
| Charlotte, NC | 214.7 | 74.9 | 34.9% | 2869 |
| Chicago, IL | 76.0 | 4.4 | 5.7% | 12155 |
| Cincinnati, OH | 265.7 | 104.4 | 39.3% | 3964 |
| Cleveland, OH | 209.7 | 8.1 | 3.9% | 4906 |
| Columbia, SC | 118.0 | 43.4 | 36.8% | 1927 |
| Columbus, OH | 170.0 | 43.0 | 25.3% | 4023 |
| Des Moines, IA | 315.7 | 49.9 | 15.8% | 3284.5 |
| Detroit, MI | 179.0 | 21.4 | 11.9% | 4732 |
| Evansville, IN | 113.7 | 13.1 | 11.5% | 2404 |
| Grand Rapids, MI | 251.0 | 30.2 | 12.0% | 3418 |
| New York, NY (Rural) | 188.0 | 75.7 | 40.3% | 2109.5 |
| New York, NY (Urban) | 399.0 | 17.5 | 4.4% | 72736 |
| Philadelphia, PA | 155.7 | 38.0 | 24.4% | 11869 |
| Washington D.C. (Rural) | 218.0 | 51.3 | 23.5% | 10018 |
| Washington D.C. (Urban) | 266.0 | 35.8 | 13.5% | 11280 |
| ^1^ Average number of samples required to reach plumes convergence across all replicates for each scenarios. ^2^ Population density obtained from US Census (2018). If >2Gy region of plume overlaps multiple sub-divisions, values were averaged. | | | | |
