# Supplementary material for: Meeting radiation dosimetry capacity requirements of population-scale exposures by geostatistical sampling
Source: PLoS One. 2020 Apr 24;15(4):e0232008. doi: 10.1371/journal.pone.0232008 (PMC7182271; doi:10.1371/journal.pone.0232008)
Supplement: S5 Table — (DOCX) [file pone.0232008.s008.docx]

| **Table S4:** **Plume Derivation with an Increased (>99%) Stringency of Overlap Between Consecutive Iterations of Geostatistical Analysis** | | | | | | | | |
| --- | --- | --- | --- | --- | --- | --- | --- | --- |
| Scenario | | | Replicate: | No. of Iterations^2^ | No. of Samples | Accuracy ≥2Gy with 90% Overlap Threshold (%) | Additional Iterations / New Unique Samples | Accuracy ≥2Gy with 90% Overlap Threshold (%) |
| City | Population Density^1^ | Conditions |  |  |  |  |  |  |
| Albany, NY | Urban | None | 1 | 3 | 106 | 75.0 | +2 / +31 | 77.4  (+2.4%) |
| Alexandria, VA | Urban | None | 1 | 4 | 137 | 57.8 | +3 / +77 | 64.5  (+7.7%) |
| Baltimore, MD | Urban | None | 1 | 4 | 111 | 75.0 | +4 / +37 | 76.2  (+1.2%) |
| Birmingham, AL | Urban | None | 1 | 4 | 98 | 67.8 | +4 / +59 | 66.3  (-1.5%) |
| Boston, MA | Urban | None | 1 | 5 | 166 | 67.7 | +2 / +43 | 65.3  (-2.5%) |
| Buffalo, NY | Urban | None | 1 | 6 | 111 | 66.8 | +2 / +107 | 68.7  (+1.9%) |
| Burlington, VT | Urban | None | 1 | 4 | 177 | 90.2 | +2 / +93 | 89.3  (-0.9%) |
| Camden, NJ | Urban | None | 1 | 5 | 136 | 71.1 | +4 /+57 | 72.2  (+1.1%) |
| Charleston, SC | Urban | None | 1 | 4 | 66 | 60.5 | +4 / +55 | 62.8  (+2.3%) |
| Charlotte, NC | Urban | None | 1 | 5 | 133 | 67.5 | +2 / +42 | 67.6  (0.1%) |
| Chicago, IL | Urban | None | 1 | 4 | 63 | 78.7 | +2 / +33 | 78.7  (-) |
| Cincinnati, OH | Urban | None | 1 | 6 | 139 | 73.2 | +4 / +63 | 73.0  (-0.2%) |
| Cleveland, OH | Urban | None | 1 | 5 | 130 | 72.0 | +3 / +87 | 72.6  (+0.6%) |
| Columbia, SC | Urban | None | 1 | 5 | 126 | 66.0 | +3 / +27 | 65.6  (-0.4%) |
| Columbus, OH | Urban | None | 1 | 4 | 74 | 66.5 | +3 / +49 | 71.6  (+5.1%) |
| Des Moines, IA | Urban | None | 1 | 4 | 240 | 74.7 | +4 / +111 | 74.3  (-0.4%) |
| Detroit, MI | Urban | None | 1 | 4 | 117 | 80.3 | +5 / +79 | 79.5  (-0.8%) |
| Evansville, IN | Urban | None | 2 | 5 | 68 | 63.4 | +4 / +23 | 63.4  (-) |
| Grand Rapids, MI | Urban | None | 1 | 9 | 199 | 72.9 | +2 / +50 | 72.7  (-0.2%) |
| New York, NY | Rural | None | 3 | 5 | 101 | 64.9 | +2 / +73 | 74.5  (+9.6%) |
|  | Urban | None | 1 | 3 | 308 | 80.3 | +3 / +85 | 81.5  (+1.2%) |
| Philadelphia, PA | Urban | None | 3 | 5 | 79 | 74.9 | +3 / +62 | 75.6  (+0.7%) |
| Washington D.C. | Rural | None | 1 | 3 | 120 | 74.7 | +4 / +126 | 75.4  (+0.7%) |
|  | Urban | None | 3 | 3 | 193 | 66.9 | +4 / +102 | 68.5  (+1.6%) |
