# Supplementary material for: Meeting radiation dosimetry capacity requirements of population-scale exposures by geostatistical sampling
Source: PLoS One. 2020 Apr 24;15(4):e0232008. doi: 10.1371/journal.pone.0232008 (PMC7182271; doi:10.1371/journal.pone.0232008)
Supplement: S1 Methods — (DOCX) [file pone.0232008.s009.docx]

**S1 Methods**

To map of predicted radiation levels (i.e. the predicted plume) based on the distribution of dose-estimated patients around the prediction location, we use kriging, a geostatistical interpolation technique which computes the spatial autocorrelation between the various data points (unlike deterministic interpolation techniques). Various kriging methods are available through the ArcMap extension “Geostatistical Analyst”, which were evaluated for this study: Ordinary, Simple, Universal, and Empirical Bayesian (Supplemental Figure 1). The general formula for kriging is:

$$Z^{'}\left( u \right)= \sum_{\alpha=1}^{n} \lambda_{\alpha}Z(u_{\alpha})$$

where $Z^{'}\left( u \right)$ is the unsampled location being predicted, $Z^{'}\left( u_{\alpha} \right)$ is the measured value, $\lambda_{\alpha}$ is the weight of measured value, and **n** is the total number of values (Leuangthong et al., 1974). Kriging is a spatially autocorrelated process which accounts for random errors, and can also be represented as:

$$Z(s) = \mu(s) + \varepsilon(s)$$

where Z(**s**) is the value of interest, µ(**s**) is the deterministic trend, and ε(**s**) are auto-correlated errors (**s** being the location of interest; <https://pro.arcgis.com/en/pro-app/help/analysis/geostatistical-analyst/what-are-the-different-kriging-models-.htm>). For simple kriging, µ is a known constant, while ordinary kriging assumes µ as an unknown constant. In universal kriging, µ(**s**) is a deterministic function (it assumes that the data has an overriding trend). Empirical Bayesian Kriging (EBK) is a method which automates the adjustment of parameters through a series of simulations. EBK uses restricted maximum likelihood (REML), while other kriging methods use weighted least squares. It also takes uncertainty into account by the estimation of the semivariogram (which characterizes the spatial autocorrelation of the data points). The main detriment to EBK is an increase in overall processing time, and can be sensitive to outliers. EBK was used for all further analyses featured in this manuscript, as the layer it created best represented the expected HPAC plume, and has the advantage of taking uncertainty measurements into account.

Other available kriging methods were not used, due to an incompatibility in data: indicator and probability kriging requires that the data be binary, while disjunctive kriging assumes the data has a bivariate normal distribution. The project data does not meet these criteria.

**Further Details on Kriging Methods**

Kriging is a geostatistical method of interpolation based on models which use spatial autocorrelation, which compares the distance between each data point. When similar data points cluster together, this is evidence of positive spatial autocorrelation (and negative when dissimilar values cluster). The following kriging methods are described in much greater detail here: [What are the different kriging models?](https://pro.arcgis.com/en/pro-app/help/analysis/geostatistical-analyst/what-are-the-different-kriging-models-.htm)

*Ordinary kriging*

- Z(**s**) = µ + ε(**s**),where µ is an unknown constant
- makes the assumption of a constant (assumption may not be true).
- aims of minimizing error variance

*Simple kriging*

- Z(**s**) = µ + ε(**s**), where µ is a known constant
- assumes that exact mean µ is known, which is often not true

*Universal kriging*

- Z(**s**) = µ(**s**) + ε(**s**), where µ(**s**) is a deterministic function.
- errors ε(**s**) are modeled to be autocorrelated
- often used in non-stationary conditions (i.e. drifting conditions)

*Indicator kriging*

- *I*(**s**) = µ + ε(**s**), where µ is an unknown constant and *I*(**s**) is binary
- meant for binary measurements (e.g. wet or dry; sun or shade)

*Probability kriging*

- *I*(**s**) = *I*(Z(**s**) > c_t_) = µ_1_ + ε_1_(**s**); Z(**s**) = µ_2_ + ε_2_(**s**); where µ_1_ and µ_2_ are unknown constants and *I*(**s**) is a binary variable created with threshold indicator (*I*(Z(**s**) > c_t_)

similar to indicator kriging, but utilizes co-kriging

- like indicator kriging, meant for binary measurements
- computing measurement error is not possible

*Disjunctive kriging*

- f(Z(**s**)) = µ_1_ + ε(**s**), where µ_1_ is an unknown constant and f(Z(**s**)) is an arbitrary function of Z(**s**).
- requires the data to have a [bivariate normal distribution](http://resources.arcgis.com/en/help/main/10.1/0031/00310000003m000000.htm)
- mathematically and computationally complicated
- computing measurement error is not possible

*Empirical Bayesian kriging*

- automatically calculates parameters through subsetting and simulations.
- uses restricted maximum likelihood (REML), unlike other kriging methods
- sensitive to outliers

**References:**

## Leuangthong,O., Khan,K.D. and Deutsch,C.V. (2008) Solved Problems in Geostatistics. Wiley-Interscience, pp.85-101.

## What are the different kriging models? 2018. Retrieved from: <https://pro.arcgis.com/en/pro-app/help/analysis/geostatistical-analyst/what-are-the-different-kriging-models-.htm>
