# Supplementary material for: Meeting radiation dosimetry capacity requirements of population-scale exposures by geostatistical sampling
Source: PLoS One. 2020 Apr 24;15(4):e0232008. doi: 10.1371/journal.pone.0232008 (PMC7182271; doi:10.1371/journal.pone.0232008)
Supplement: Attachment — Submitted filename: Response to Reviewers.docx [file pone.0232008.s010.docx]

Gayle E. Woloschak, PhD
Academic Editor
PLOS ONE

Dear Dr. Woloschak:

Thank you for allow us to revise our manuscript for PLoS ONE. We appreciate your consideration of the revised manuscript. We are grateful for the insightful comments by the Referees, which we have address in the revision and respond to below.

Please note that we have added one author, Ben C. Shirley, who has contributed software development expertise to this work.

Kind regards,

Peter K. Rogan, Ph.D. (on behalf of all of the authors)

University of Western Ontario

**Response to Reviewers**

**Comments to the Author**

1. Is the manuscript technically sound, and do the data support the conclusions?

The manuscript must describe a technically sound piece of scientific research with data that supports the conclusions. Experiments must have been conducted rigorously, with appropriate controls, replication, and sample sizes. The conclusions must be drawn appropriately based on the data presented.

Reviewer #1: Partly

Reviewer #2: Yes

Since Reviewer #1 did not indicate specific issues, we are unable to address their response.

3. Have the authors made all data underlying the findings in their manuscript fully available?

The [PLOS Data policy](http://www.plosone.org/static/policies.action#sharing) requires authors to make all data underlying the findings described in their manuscript fully available without restriction…

Reviewer #1: No

Reviewer #2: Yes

In response to reviewer #1, we have uploaded the HPAC derived data and our scenario sample locations with inferred radiation levels for each scenario to a Zenodo.archive (<https://doi.org/10.5281/zenodo.3572574>). The address of this archive is also indicated in the manuscript.

5. Review Comments to the Author

Please use the space provided to explain your answers to the questions above. You may also include additional comments for the author, including concerns about dual publication, research ethics, or publication ethics. (Please upload your review as an attachment if it exceeds 20,000 characters)

Reviewer #1:

A) This manuscript has two main elements – one good and one bad. First, the methodology of geostatistical sampling and guidance of future, iterative sampling is logical and well-described. It could have great utility in a scenario of limited data acquisition and processing to replace universal assessment (impossible) with a plan to most rapidly and efficiently acquire the necessary limited data to refine (and confirm) a priori models. However, the application of this approach to the second element – biodosimetry via cytogenetic assessment of dicentric chromosomes – appears to be without merit or value. Also, it is unnecessary. For all the work, the authors have simulated biodosimetry data from physical dose values, with the assumption that the latter is the ground truth. Essentially, this means they could have removed the entire aspect of biodosimetry and simply applied the geostatistical sampling approach to the question of where to focus a limited number of physical dose assessments so as to confirm/modify predicted models of the radiation plume. So they have basically made a circular argument to go from physical dose to biological dose to arrive at agreement with models of physical dose.

We have modified the text of the manuscript in order to generalize the source of radiation measurement to either physical or biodosimetry methods. While the method was conceptualized with biodosimetry in mind, the method would still be useful to reduce physical sampling requirements in population-scale radiation scenarios and would be expected to decrease total radiation exposures of first responders. We have generalized the text to avoid specifying a particular dosimetry method (e.g. “cytogenetic biodosimetry” changed to “dosimetry”). As this transition required numerous edits (too many to describe here), we have provided select examples of where the text has been modified to include physical dose measurement:

1) Abstract (Background section):

“Even with high-throughput dosimetry analysis, test volumes far exceed the capacities of first responders to measure radiation exposures directly, or to acquire and process samples for follow-on biodosimetry testing.”

2) Abstract (Results/Conclusions section): “Geostatistical mapping limits the number of individuals requiring **laboratory testing**, the time required, and radiation exposure to first responders.”

This has been modified to (see **bolded t**ext):

“Geostatistical mapping limits the number of individuals requiring **dose assessment**, the time required, and radiation exposure to first responders.”

3) Introduction (Paragraph 4, page 4): “The question this paper addresses is whether the radiation plumes derived by HPAC can be reconstructed with kriging using a relatively small number of samples analyzed by physical or biodosimetry methods.”
4) Results (Paragraph 1, page 11): “We simulated the analysis of 28 population-scale, 10 megaton yield nuclear detonation scenarios with ADCI-HT using cytogenetic biodosimetry data…”

This has been modified to:

“We simulated the analysis of 28 population-scale, 10 megaton yield nuclear detonation scenarios with dosimetry data…”

5) We have also greatly modified the Introduction paragraph describing dosimetry methods to expand information regarding physical dose methods. Example:

Introduction (Paragraph 2, page 3): “Physical dose can be inferred from the prodromal response of absorbed radiation, which include erythema, headache, fever, lethargy, tachycardia, nausea and vomiting (1,3).”

However, results from biodosimetry can be achieved in a clinically relevant time frame. The estimates given in the manuscript were simply examples based on certain assumptions about laboratory capacity. With laboratory scale-up and parallelization (e.g. commercial cytogenetic laboratories with highly automated sample preparation and redundant microscope imaging systems), the time to determine and report absorbed radiation would be reduced significantly, e.g. 3-4 days. This approach has been suggested as part of the U.S. Concept of Operations in a nuclear incident, which is now cited in this article as reference 36. We previously suggested this possibility at a BARDA TechWatch presentation in 2012 and provided relevant data to these authors (who presented it at the most recent EPR Biodose conference in Munich, 2018). As the text has been modified to include physical dosimetry methods, the majority of text discussing cytogenetic biodosimetry has been moved to the latter part of the Discussion (pages 18-20).

We carefully describe the assignment of dose values in the simulation to demonstrate that the results presented are not the result of cyclical reasoning. The HPAC plume is considered the “ground truth” dose estimate, however the actual portable data generated and exported by the HPAC software is confined to the values at the contours that make up the plume itself. Locations between the geospatial coordinates given by the contours are not provided by HPAC. For more accurate dose estimation, these values should be interpolated between adjacent contours. To avoid a circular reasoning argument we have not performed such interpolation, rather we use the dose estimate of the outer contour adjacent to sampled locations (regardless of whether they intersect the contour or not), that is, we introduce a source of systematic error to avoid the circular argument. The data collected by first responders after kriging and densification should reflect the actual dose at the site sampled. When we then compare the derived plume with the HPAC plume, we are comparing the dose that was derived from the corresponding interval separated by contours with what is expected from geostatistical estimation of dose. The goal was to demonstrate that this approximation method provides actionable information about exposures based on the sampling procedure, and that we can reconstruct the plume from a small fraction of the samples that were used to generate it (<10%). Biological dose estimation for large scale events with geolocation data equivalent to HPAC models has not been previously reported. Without such a map, it is not possible to directly compare bio- with physical dose estimates for a large-scale incident. We therefore simulate the biological dose by sampling the HPAC map at many fewer locations that HPAC uses to derive plume contours.

B) A second major problem with the application to biodosimetry deals with the re-iterative process. Identification of sites/individuals for subsequent sampling works when the sampling is not a bottleneck – both in terms of capacity (number that can be processed at any time) and throughput (time for generation of the next set of data). The first is obvious and pointed out by the authors – with examples of 160 to 400 samples being presented as possible realistic goal for a “round” of assessments. From this, one-two rounds of testing would be required to provide the necessary samples (Table 1). But, one does not know at start where to sample for each iteration. And one cannot start subsequent sampling until the initial model is produced. With a turn-around time of 1.5 weeks, a model requiring 3-4 iterations would be worthless in terms of timely provision of information on how to triage and who to treat. Even if all bottlenecks are not consecutive, one still has minimum of a week from sampling to a data point (culturing, preparing metaphase cells, and imaging) before the next round can start. Further, if the situation of dose accumulation is not static – which the authors admit it is not – then the data from samples collected in subsequent iterations may not really extend the data in the first iteration. This makes the whole application unfeasible. In contrast, sticking to physical dose assessments, the turn-around time between iterations might be only one day and thus a feasible approach to rapid and efficient real dose-mapping to confirm models.

We have added text to address where first-responders could perform initial sampling (Methods section, first paragraph, page 4):

“Initially, locations within likely fallout areas were sampled from the potentially affected population. In a real-world scenario, initial randomly sampled locations would be selected based on approximate wind direction and location of the epicenter (“ground zero”).”

We have also made significant changes throughout the text in order to generalize the potential sources of radiation measurements to include physical dose. The methodology proposed in this manuscript should be feasible regardless of the dosimetry method used to derive it. For any radiation metric, the proposed approach should benefit first responders by reducing sampling requirements, and consequently their exposure to radiation.

Regarding your comments about cytogenetic biodosimetry turnaround time, please see our comment in part (A).

C) There is also a problem with the language used in several places. While the whole concept is one of limited sampling to aid in prediction modeling, there are statements that imply universal assessment of all potentially irradiated individuals. Last sentence of first paragraph on page 17 states that a capacity of 400 samples “should be sufficient to diagnose most or all individuals with symptoms of Acute Radiation Syndrome”. First, this has nothing to do with diagnosis, only assignment of a biological dose (>2 Gy) that carries with it a potential for ARS. And with actual symptoms of ARS (a diagnosis) one probably needs nothing further to initiate treatment.

Thank you for your suggestion. We have corrected any text that describes Acute Radiation Syndrome (ARS) to make it clear that a >2Gy dose only indicates the potential risk of ARS and is not a diagnosis of ARS. For example, the sentence you indicated on page 17 (now on page 19) is now written as follows:

“Timely and accurate assessment will be needed to inform health professionals about individuals at high-risk for Acute Radiation Syndrome, preferably during the prodromal phase.”

Another example where we discussing a method to expedite identification of those at risk for ARS, not diagnosis of ARS:

Introduction (paragraph 1, page 3): “Individuals at risk for acute radiation syndrome, e.g. those receiving ≥ 2Gy exposure, should be rapidly identified by these approaches to determine eligibility for therapy (2).”

D) Further, the final prediction of biological dose locations (contours) at best will confirm a physical dose map derived from either actual dose measurements or the HPAC models. But what if it doesn’t? Either because there is not a one-to-one correlation between biological dose and physical dose (again, the authors acknowledge this) or because the model is not applicable. And the more “discrepancies” one has in the first iteration, the harder it will be to arrive at the final mapping.

The manuscript already addresses this issue. We have explicitly introduced the possibility that the deviation could involve physical vs biodosimetry dose estimation:

Results (final paragraph, page 16): “Maximum deviations were designed to represent confidence values in physical and/or biodosimetry methods, including dicentric analysis (DCA; ±0.5Gy) or cytokinesis-blocked micronucleus assays (±1.0Gy), which exhibit higher variance in estimating dose compared to DCA (28–30). The introduction of such measurement errors led to plumes that were generally less accurate, required a greater number of samples, and additional iterations of kriging and densification to achieve convergence (S2 Table). The degree of error in this analysis was also correlated with decreased accuracy and increased processing time (±0.5Gy error-derived plumes were more accurate than ±1.0Gy error-derived plumes in nearly all scenarios; S2 Table).”

Note that while an increase in dosimetry error both increases sampling requirements and modestly decreases overall accuracy, the method will still ultimately derive a plume regardless.

E) In the end, the use of geostatistical sampling, at least when applied to limited biodosimetry data, is unlikely to be “one of the only practical solutions in a radiation mass casualty to quickly and accurately triage large populations for therapeutic decisions”. It will not be quick – in time to initiate treatment for those who might potentially benefit. And there will be no way to determine how accurate it is.

We have altered this statement:

Discussion (paragraph 7; page 20): “Geostatistical sampling may provide a practical solution to quickly triage these exposures in large populations.”

There are promising approaches to expedite the preparation of samples and data acquisition steps (Dainiak et al. 2019; Ref. #36) so that triage biodosimetry could be carried out sufficiently quickly to benefit many of those requiring treatment. Furthermore, we have broadened the scope of the paper to include reconstruction of physical radiation plumes from geostatistically sampled subsets of locations or individuals. Reconstruction of the plume could be carried out within a few minutes if the radiation detectors are placed at geolocations specified by kriging and densification.

**Reviewer #2:**

This is an interesting exercise on an attempt to map radiation plumes and fallout to determine a sample of individuals to test for radiation exposure in the case of a nuclear event. There are several concerns around this manuscript.

1. it is not clear how much better this will be compared to simple dosimeters that are commonly found in cities. mapping this mathematically does not appear to add much to the data that is possible to be collected by physical biodosimetry.

In the Introduction, we cite previous studies that illustrate inaccuracies when comparing results of physical dosimetry and cytogenetic biodosimetry for the same individuals from the same exposure. Such discrepancies have implications for medical emergency management:

Introduction (Paragraph 2, page 3): “Confounding factors may affect testing results. These include the possibility of discordant physical radiation measurements and estimated biodosimetry exposures (13,14), and other causes of indirect predictors of exposure such as time-to-emesis (such as head trauma, or comorbid infections), thereby reducing accuracy of diagnoses (15).”

The manuscript has been modified to put a greater emphasis on the benefits of this method for reconstructing exposures over affected geographic areas for any type of dosimetry, including physical dosimetry (see our response to Reviewer #1 [A]). Despite physical dosimetry being much faster compared to cytogenetic biodosimetry, determining the physical dose of all individuals in a large city is still infeasible. In the early stages of a nuclear event, collecting data would be difficult for first responders. In this situation, the proposed approach would allow for the development of an accurate radiation plume with only limited sampling while decreasing exposure of first responders by minimizing time in irradiated areas.

2. the major concern is that the radiation doses in a large city will be inhomogeneous due to partial shielding. How does this algorithm account for such events?

The third paragraph of the Discussion describes the limitations of our method. Here, we discuss how shielding (or “shadowing”) by infrastructure is not accounted for, and how these concessions may limit the overall accuracy of our method.

Results (Paragraph 3, page 18): “Finally, neither the HPAC version available for this study, nor our geostatistical models account for shielding by infrastructure, such as shadowing, which computes the degree to which radiation is prevented from reaching certain locations by the urban environment (34). These concessions may limit the overall accuracy of radiation measurements that we are using as the ground truth.”
