# Supplementary material for: Meeting radiation dosimetry capacity requirements of population-scale exposures by geostatistical sampling
Source: PLoS One. 2020 Apr 24;15(4):e0232008. doi: 10.1371/journal.pone.0232008 (PMC7182271; doi:10.1371/journal.pone.0232008)
Supplement: Attachment — Submitted filename: Response to Reviewers 2.docx [file pone.0232008.s011.docx]

**Academic Editor Comments**

We would appreciate receiving your revised manuscript by Feb 13 2020 11:59PM. When you are ready to submit your revision, log on to <https://www.editorialmanager.com/pone/> and select the 'Submissions Needing Revision' folder to locate your manuscript file.

If you would like to make changes to your financial disclosure, please include your updated statement in your cover letter.

To enhance the reproducibility of your results, we recommend that if applicable you deposit your laboratory protocols in [protocols.io](http://protocols.io/), where a protocol can be assigned its own identifier (DOI) such that it can be cited independently in the future. For instructions see: <http://journals.plos.org/plosone/s/submission-guidelines#loc-laboratory-protocols>

**Author Response (Cover letter)**

Dear Dr. Woloschak:

Thank you for allowing us to once again revise our manuscript for PLoS ONE. We have thoughtfully considered the comments made by the first referee, and we have responded to each point in a separate letter provided below. As you recommended, we have also included a detailed protocol for generation and comparison of radiation dosimetry maps using the method described in the paper and uploaded this protocol to [protocols.io](https://www.protocols.io/) (<http://dx.doi.org/10.17504/protocols.io.ba4nigve>). This protocol has been named "Protocol for Geostatistical Determination of Radiation Dosimetry Maps of Population-Scale Exposures".

The first referee expresses their specific point of view regarding the protocols and guidelines for application of physical dosimetry vs biological dosimetry methods and relegates biological dosimetry to a secondary role. Recent advances in these techniques, in particular interpretation of data, have demonstrated that timely and useful information can inform clinical decisions. We never intended our manuscript to be a discourse on benefits vs. drawbacks of performing different types of dosimetry. Discussion of preference between physical and bio-dosimetry methods is not relevant to the scientific validity of the work, which is one of the reasons why we submitted our manuscript to PLoS ONE.

The point of our manuscript is to introduce a new method of deriving accurate radiation plumes requiring far fewer direct measurements than previously thought necessary. While plumes can be derived with the geostatistical approach described in our manuscript using any dosimetry method, it has particular implications for biodosimetry, which has been previously dismissed by many (including this reviewer) as too slow to be used to guide management of radiation-caused illness. Rather, maps derived from biological radiation measurements that significantly exceeding the recognized threshold of 2Gy are likely precise enough to begin treatment prior to obtaining the final plume.

Putting the issue of the benefits or drawbacks of performing different types of dosimetry aside, we hope that you find our responses to the first reviewer’s comments acceptable; and that you consider our manuscript for publication in PLoS ONE.

Kind regards,

Peter K. Rogan, Ph.D. (on behalf of all of the authors)

**Reviewer comments (Author Responses follow each comment)**

1. If the authors have adequately addressed your comments raised in a previous round of review and you feel that this manuscript is now acceptable for publication, you may indicate that here to bypass the “Comments to the Author” section, enter your conflict of interest statement in the “Confidential to Editor” section, and submit your "Accept" recommendation.

Reviewer #1: (No Response)

Reviewer #2: All comments have been addressed

2. Is the manuscript technically sound, and do the data support the conclusions?

The manuscript must describe a technically sound piece of scientific research with data that supports the conclusions. Experiments must have been conducted rigorously, with appropriate controls, replication, and sample sizes. The conclusions must be drawn appropriately based on the data presented.

Reviewer #1: Partly

Reviewer #2: Yes

RESPONSE

Reviewer #1 has misunderstood the results by confusing the “iterative” process of kriging and densification with the analysis of 3 completely independent replicates of scenarios initiated using different random seed conditions (see Section 6.3 below). We have submitted a detailed protocol to the [protocols.io](https://www.protocols.io/) website that demonstrates this iterative process. We have also uploaded the sample data for each replicate of each scenario, along with project-related software that are used with ArcMap to perform the geostatistical analyses to the public repository, Zenodo. Reviewer #1 is invited to review these resources and reproduce our findings prior to drawing conclusions regarding their adequacy.

3. Has the statistical analysis been performed appropriately and rigorously?

Reviewer #1: No

Reviewer #2: Yes

RESPONSE

There is no factual basis for this conclusion by Reviewer #1. We emphatically deny that we have removed or selectively altered any data presented in this study. In fact, the reviewer’s conclusion appears to have been based on a misinterpretation of the results obtained for the Columbia SC scenario (see Section 6.3 below) alone. The statistical analyses for all 24 scenarios have been performed appropriately and rigorously, according to accepted methods in geostatistical analyses.

As mentioned in our previous response, we have uploaded the HPAC-derived data and our scenario sample locations with inferred radiation levels for each scenario to a Zenodo archive (<https://doi.org/10.5281/zenodo.3572574>). In addition to this, we also include a detailed protocol for generation and comparison of radiation dosimetry maps using the method described in the paper and uploaded this protocol to protocols.io ([dx.doi.org/10.17504/protocols.io.ba4nigve](https://www.protocols.io/view/protocol-for-geostatistical-determination-of-radia-ba4nigve)). The protocol contain links to data and programs in the Zenodo archive, however these links will not be live until the archive is formally published. This will occur should the paper be acceptable for publication by PLoS ONE.

4. Have the authors made all data underlying the findings in their manuscript fully available?

The [PLOS Data policy](http://www.plosone.org/static/policies.action#sharing) requires authors to make all data underlying the findings described in their manuscript fully available without restriction, with rare exception (please refer to the Data Availability Statement in the manuscript PDF file). The data should be provided as part of the manuscript or its supporting information, or deposited to a public repository. For example, in addition to summary statistics, the data points behind means, medians and variance measures should be available. If there are restrictions on publicly sharing data—e.g. participant privacy or use of data from a third party—those must be specified.

Reviewer #1: Yes

Reviewer #2: Yes

5. Is the manuscript presented in an intelligible fashion and written in standard English?

PLOS ONE does not copyedit accepted manuscripts, so the language in submitted articles must be clear, correct, and unambiguous. Any typographical or grammatical errors should be corrected at revision, so please note any specific errors here.

Reviewer #1: Yes

Reviewer #2: No

RESPONSE

We do not understand this conclusion by reviewer 2, who has recommended acceptance of the manuscript. Elaboration is requested.

**6. Review Comments to the Author**

Please use the space provided to explain your answers to the questions above. You may also include additional comments for the author, including concerns about dual publication, research ethics, or publication ethics. (Please upload your review as an attachment if it exceeds 20,000 characters)

Reviewer #1:

1. While substantial improvements have been made, there is still a remaining problem that will mislead a naïve reader. That is the continued emphasis on ”individuals” that implies more than is warranted. While the authors have made the necessary modifications to replace the biological dosimetry with physical dose measurement, there is still text that implies assessment of individuals (i.e., biological dosimetry) rather than locations (physical dosimetry). Although the simulated measurements based on location can be weighted for population density, it still does not provide information at the individual level.

RESPONSE

The method described in this manuscript would allow for the derivation of a radiation plume using any (consistent) type of radiological measurement. This includes the physical measurement of radiation of a location, but this also includes biological dosimetry of individuals, which is what we are referring to here. The Introduction describes how first responders and medical personnel have qualitatively inferred exposures based upon presentation of symptoms, aside from simply obtaining direct measurements via Geiger counters:

“Physical dose can be inferred from the prodromal response of absorbed radiation, which include erythema, headache, fever, lethargy, tachycardia, nausea and vomiting (1,3).”

To eliminate any confusion, we have added text to the method which indicates that “sample” can refer to any radiological measurement type:

Methods: “…we refer to these locations as samples, regardless of whether the radiation is quantified from either physical emissions or from absorbed biological effects. We assume that all measurements used to derive the plume are consistently obtained by the same approach.”

Biodosimetry of individuals can provide actual exposure measurements, whereas physical dosimeters measure environmental radiation emission levels. While physical dosimetry measurement is more rapid than biodosimetry, there are limitations in applying unfiltered data to derivation of radiation plumes. There are well known false positive readouts obtained through aerial physical dosimetry counterterrorism surveys (Karam PA, “Radiation in Daily Life,” ANS Nuclear Cafe website; Aug. 28, 2017):

<http://ansnuclearcafe.org/2017/08/28/radiation-in-daily-life/#sthash.pSWLh6N3.dpbs>)

Common environmental sources of radiation, such as large deposits of granite (i.e. headstones in cemeteries), as well as medical and construction sites (i.e. radiation from poorly maintained X-Ray machines), can lead to false positive spikes in physical radiation unrelated to the nuclear incident that we are attempting to model using multiple measurements dispersed across the landscape. If used, these data could distort a plume derived by geostatistical analysis. These factors would be somewhat mitigated by depositing physical detectors at ground locations specified by this iterative densification, as suggested in the paper. Nevertheless, it would appear that there are certain advantages of performing biodosimetry on individuals at specified locations, as an alternative to surveying locations themselves for radioactive emissions.

2. An example of the focus on individuals is a phrase that is repeated in the Abstract (Results) and the first paragraph of the Discussion:
On average, 71±9% of those with ≥2Gy exposures were accurately localized.
plumes from 28 distinct scenarios simulating absorbed radiation identified 71±9% of individuals with ≥2Gy exposures
A better phrasing would be “71±9% of those locations where the population might be expected to receive an exposure greater than 2 Gy were identified.”

RESPONSE

The value of 71±9% accuracy has been corrected (to 70±10%) in the latest version of the manuscript. As the paper states, each scenario initially consisted of three replicates, however we did perform additional replicates for 2 of the scenarios which exhibited larger inter-replicate variation in performance. An additional 2 replicates for these scenarios were included close to the completion of the manuscript. Inclusion of these additional replicates (Columbia SC replicates #4 and 5, and the Columbus OH replicates #4 and 5) resulted in a minor adjustment to the accuracy calculation, which was not statistically different from the results based on 3 replicates. The omission of the additional replicates from the accuracy calculation was inadvertent.

We appreciate the reviewer’s suggestion, and have made said change to the manuscript, with slight adjustments:

Abstract: “On average, 70±10% of locations where populations are expected to receive an exposure ≥2Gy were identified.”

Discussion: “Overall, plumes from 28 distinct scenarios simulating absorbed radiation identified 70±10% of locations where the population are expected to receive ≥2Gy of radiation exposure (and thus are eligible for cytokine therapy), …”

Furthermore, the overall accuracy of 70.6% has been adjusted to 69.8%.

3. There is also a problem with how the central data in Table 1 are interpreted. As stated on page 13, “Success” was based on accuracy of predicting irradiated samples greater than 2 Gy. The authors took the final iteration values and stated an average accuracy of 70.6%. But this is after omitting an outlier for Columbia (or replacing with a further re-iteration attempt – it is not clear which).

There was also an intermediate outlier for Columbus but the final value was in range and was apparently used. No mention is made of Charleston where the final value exceeds the 10% threshold of difference from either of the first two values. Most importantly, the average accuracy from the final values after the process is no different from the average accuracy computed using the first iteration values. Just as many locations had a decrease from first to final iteration as had an increase. And excluding the 10% increase for Charleston the maximum “improvement” was 4% while the maximum loss of accuracy was 5%. So, what is the value of this iterative process? While it may have the potential to refine predicted dose maps, it clearly does not provide better results for “success” based on the accuracy values.

RESPONSE

This was a misunderstanding by the reviewer, since outliers were not excluded at any time in any computation. The final accuracy for each of the replicates (replicate #1, 2 and 3) were included in the computation of the overall accuracy of the project. As previously mentioned, inclusion of replicates #4 and 5 for Columbia SC and Columbus OH slightly decreased this value (from 70.6% to 69.8%).

There also appears to have been a fundamental misunderstanding of the results presented in Table 1. In contrast to the assertion by the reviewer, replicates shown in Table 1 are not a step-by-step description of the progression of our iterative process. Instead, they each represent independently-derived plumes (each consisting of multiple iterations of kriging and densification) differing only in their starting set of random determined sample locations. The replicates are arbitrarily labeled as numbers 1,2 and 3. *These replicates are not presented in any particular order.*

From the Methods:

“Comparisons between independent replicates of the same scenarios using different, randomized, initial sample distributions evaluated the reliability of this approach.**”**

**“**The geostatisical workflow was used to analyze three replicates of each scenario, with each replicate initiated with a different set of random sample locations within the plume.”

The plumes derived from the initial set of points alone are less accurate than the final plume. In low population-density or sparsely sampled regions, the HPAC and derived plumes show limited overlap of the same contours resulting in poor accuracy for defining the 2Gy threshold. Neither example in Figure 2 exhibits this issue because of the relatively higher sampling densities in these scenarios. Reduction in the off-diagonal cell values over consecutive iterations in the tables below the plume maps illustrates the improvement in the model. We do not believe that it would be as informative to the reader to compute accuracy metrics between iterations, and generally only present accuracy at the end of the procedure (except for the new Columbia SC replicate shown below).

Nevertheless, we address the reviewer’s critique of our results for the Columbia SC scenario by performing this analysis for a new, previously unreported replicate. The following results are now reported as an example of the procedure (at <https://www.protocols.io/>) described in the Methods of our manuscript.

We find:

- Accuracy of plume from initial set of random samples only: **0% accuracy** (the entire plume is <2Gy; image of plume in Section 4 of the [online protocol](https://www.protocols.io/view/protocol-for-geostatistical-determination-of-radia-ba4nigve)) [N=8 samples > 0 Gy]
- Accuracy after 1 Iteration: **21.1% accuracy** (image of plume in Section 5 of the [online protocol](https://www.protocols.io/view/protocol-for-geostatistical-determination-of-radia-ba4nigve)) [N=55 samples > 0 Gy]
- Accuracy after 3 Iterations: **46.0% accuracy** [N=79 samples > 0 Gy]
- Accuracy after 5 Iterations: **68.4% accuracy** (final derived plume; image of plume in Section 6 of the [online protocol](https://www.protocols.io/view/protocol-for-geostatistical-determination-of-radia-ba4nigve)) [N=136 samples > 0 Gy]

As this was a source of confusion, we have added a new comment in the footnote to Table 1 for the “No. of Iterations” column:

“^2^ The number of iterations (kriging and densification steps) required to reach stopping criteria for this replicate.”

Additionally, in places in the manuscript where we discuss the iterative process, we have added text to make it clear that we are referring to iterations of kriging and densification. For example:

Methods (new text bolded): “These **kriging and densification processes are** repeated for a limited number of iterations until the coverage area and the radiation level contours of the inferred plume stabilizes”

4. Finally, despite the shift from biodosimetry to physical dose sampling, there is still the underlying implication that this process may be applied to “a relatively small number of samples analyzed by physical or biodosimetry methods” (last sentence of Introduction). Similarly, on page 20, “Population-scale radiation exposure identification can be achieved through a combination of high-throughput dicentric chromosome identification software and GIS-based software analysis, and the test volume is likely to be feasible for a large dosimetry lab.” Any aspect that relies on an iterative process using cytogenetic data is patently not feasible for triage purposes when there is a minimum 1 week turn-around time. One might use the reiterative process on physical dose mapping to select locations where selected individuals are then assessed by biodosimetry – but this is not the take-away message from the manuscript as written.

RESPONSE

We have not “shifted the emphasis” of this paper to sampling by physical dosimetry. Rather, we have expanded our approach to include sample data originating from either physical- or bio-dosimetry. The approach we describe can be performed using either type of data.

Biological dosimetry would still be feasible to generate meaningful plumes, leading to sufficiently timed treatment. The 1.5-week scenario that we describe in the Discussion accounted for all iterations of the plume development process and assumed that a single or two typical biodosimetry laboratories would perform the sample processing and analysis. The revised manuscript cited a recent Concept of Operations study (Daniak et al. 2019) that envisions engagement by large commercial cytogenetic operations with substantial automation of these processes. We have attempted to make this clearer in the text (new text bolded):

“The expected throughput of cytogenetic biodosimetry of a typical small city scenario (~160 samples) is less than 1 week **(for all iterations per scenario, cumulatively)**, assuming…”

While sample culturing introduces an initial 48-hour lag, the period afterwards would be quite rapid due to A) automated sample preparation, B) multiplexed imaging of samples in parallel, C) automated dicentric chromosome detection and radiation dose estimation, and D) kriging and densification. Additional samples can be collected during some of these analysis steps. We envision a continuous process, not a segmented one as the reviewer has suggested.

Geographic regions close to the epicentre receiving high levels of radiation exposures (> 3Gy) will be evident early in the process. It should not be necessary to wait until the final iteration is completed to apply this information in clinical decision making. We have not investigated this possibility in detail in the current manuscript, however we plan to examine this possibility in future studies. If proven, it may be feasible to consider eligibility for therapy using the plume derived after the first iteration of geostatistical analysis. While the 2Gy contour may not be well defined after a single iteration, we note that locations where absorbed radiation exposures are highest generally cover the similar geographic contours in subsequent plumes. Individuals in imminent need of treatment would benefit sooner, while management of those with lower, clinically relevant radiation exposure would be deferred until the plume is better defined.

It is not our intent to evaluate or compare physical versus biological dosimetry methods in this study. The purpose was to introduce a geostatistical method to deriving radiological plumes from any measurement source. Biodosimetry covers many techniques, however our work has primary focused on advancing the state-of-the-art of the dicentric chromosome assay (see <https://adciwiki.cytognomix.com/doku.php?id=main:references>). The Discussion of this paper therefore describes the impact of the geostatistical approach on this assay. Other, more rapid biodosimetry assays could also be used to derive a plume based on absorbed radiation, however the dose estimates obtained with these techniques have lower confidence. We also present results addressing the impacts of less accurate measurements on derived radiation plumes, whether they are derived from biological or physical dosimetry data.
