# Supplementary material for: Meeting radiation dosimetry capacity requirements of population-scale exposures by geostatistical sampling
Source: PLoS One. 2020 Apr 24;15(4):e0232008. doi: 10.1371/journal.pone.0232008 (PMC7182271; doi:10.1371/journal.pone.0232008)
Supplement: Attachment — Submitted filename: The authors use geospatial modeling in an attempt to decrease the need for radiation dosimetry in the setting of a large scale disaster.docx [file pone.0232008.s012.docx]

The authors use geospatial modeling in an attempt to decrease the need for radiation dosimetry in the setting of a large scale disaster. They modeled 30 scenarios, including 22 urban/high-density and 2 rural/low-density scenarios under various weather conditions. Multiple (3-10) rounds of sampling and kriging were required for the dosimetry maps to converge, requiring between 73 and 417 samples for different scenarios. On average, 70±10% of locations where populations are expected to receive an exposure ≥2Gy were identified.

They conclude that geostatistical mapping limits the number of individuals requiring dose assessment, the time required, and radiation exposure to first responders. Geostatistical analysis will expedite triaging of acute radiation exposure in population-scale nuclear events.

This manuscript is a highly mathematical approach to a major problem in a mass casualty event concerning radiation exposure. The major hurdle with this reviewer is that this approach does not really solve the problem. Based on their data from table 1, the range is so variable that this reviewer does not see the value of this approach. For example, the accuracy is as low as in the 20% range making this approach inferior to a coin toss. The best case scenario is in the low 80% range. I am not clear that this provides much more information since it’s not clear what the responders would approach the other 20% that did receive a higher dose.

Lastly, a major difficulty is partial body shielding which would make this approach even more difficult to interprete.
