# Supplementary material for: Meeting radiation dosimetry capacity requirements of population-scale exposures by geostatistical sampling
Source: PLoS One. 2020 Apr 24;15(4):e0232008. doi: 10.1371/journal.pone.0232008 (PMC7182271; doi:10.1371/journal.pone.0232008)
Supplement: Attachment — Submitted filename: Response-to-Reviewers.Third-Revision Final.docx [file pone.0232008.s013.docx]

Summary of New Data Analyses added to Version 3 of the manuscript:

1. We computed the accuracy of the derived plumes at the 3Gy radiation level. We hypothesized that because regions more distant from the epicentre of the nuclear event were less densely populated and less compact at the 2 Gy contour compared to the 3 Gy contour in some scenarios that the performance of the proposed method might improve at the higher radiation threshold. Plumes at the 3Gy level were similar to the HPAC plume in many scenarios (see Table 1, rightmost column), and this is described in the Results.
2. The initial conditions for stopping our procedure (>90% concordance between areas of consecutive plumes) were specified for rapid computation of an initial radiation plume map in a triage situation. We determined whether these convergence criteria were optimal using a higher stringency of overlap between the plumes of consecutive iterations of geostatistical analysis (>99% concordance) for one replicate in each scenario. The accuracy for some scenarios improved significantly (supplementary table S4, Figures S2 and S3), and this is summarized in the Results.
3. We estimated the magnitude of 2 Gy plume error by varying the contour threshold of the derived plume until the differences between the HPAC and derived plumes were minimized. Based on Reviewer #2 comments, a significant amount of the error reported for each scenario replicate is relatively close to the desired threshold. For example, 63% of Boston MA replicate #3 is accurate at the 2Gy contour, but 86% of the missing plume area occurs within 1.7-2.0 Gy exposure level. This is now described in the Discussion.

**Comments to the Author**

1. If the authors have adequately addressed your comments raised in a previous round of review and you feel that this manuscript is now acceptable for publication, you may indicate that here to bypass the “Comments to the Author” section, enter your conflict of interest statement in the “Confidential to Editor” section, and submit your "Accept" recommendation.

Reviewer #1: (No Response)

Reviewer #2: All comments have been addressed

2. Is the manuscript technically sound, and do the data support the conclusions?

The manuscript must describe a technically sound piece of scientific research with data that supports the conclusions. Experiments must have been conducted rigorously, with appropriate controls, replication, and sample sizes. The conclusions must be drawn appropriately based on the data presented.

Reviewer #1: Partly

Reviewer #2: No

 Response:

We note that Reviewer #2 has changed their decision from Yes in the previous two revisions of the manuscript. Based on Reviewer #2 comments in this most recent review, we assume this is due to the accuracy values presented in the manuscript, which have now changed since the initial draft of the manuscript. The particular replicates that the Reviewer has commented on consist of small numbers of samples in two scenarios with low density populations predominantly in rural areas. We have addressed this by performing additional simulations with increased initial population densities (0.2, 1.0%) and adding additional iterations of kriging and densification. Both of these have improved the final accuracies for these replicates.

3. Has the statistical analysis been performed appropriately and rigorously?

Reviewer #1: Yes

Reviewer #2: Yes

4. Have the authors made all data underlying the findings in their manuscript fully available?

The [PLOS Data policy](http://www.plosone.org/static/policies.action#sharing) requires authors to make all data underlying the findings described in their manuscript fully available without restriction, with rare exception (please refer to the Data Availability Statement in the manuscript PDF file). The data should be provided as part of the manuscript or its supporting information, or deposited to a public repository. For example, in addition to summary statistics, the data points behind means, medians and variance measures should be available. If there are restrictions on publicly sharing data—e.g. participant privacy or use of data from a third party—those must be specified.

Reviewer #1: Yes

Reviewer #2: Yes

5. Is the manuscript presented in an intelligible fashion and written in standard English?

PLOS ONE does not copyedit accepted manuscripts, so the language in submitted articles must be clear, correct, and unambiguous. Any typographical or grammatical errors should be corrected at revision, so please note any specific errors here.

Reviewer #1: Yes

Reviewer #2: No

Response:

Documentation of specific errors as requested by PLOS ONE has not been provided by Reviewer #2.

Based on the evidence after multiple rounds of review, reviewer #2 clearly understands the main points addressed in the manuscript, that is, the paper is written in an intelligible fashion. After multiple revisions, this Reviewer has not listed any errors in our grammar, spelling or clarity of presentation.

**6. Review Comments to the Author**

Please use the space provided to explain your answers to the questions above. You may also include additional comments for the author, including concerns about dual publication, research ethics, or publication ethics. (Please upload your review as an attachment if it exceeds 20,000 characters)

Reviewer #1:

1. This manuscript is definitely getting better; however, there remain problematic issues that relate to the potential for a causal reader to be misled as to what was done and what the results mean.

Response:

On multiple occasions, this Reviewer has indicated that we have mislead or have the potential to mislead the casual reader regarding terminology related to the types and methods of processing samples. We do not share this view. Nevertheless, have modified our manuscript to be agnostic about the type of dosimetry that can be used with the method we propose. We sincerely believe that this latest revision alleviates any remaining issues that led to their perception. We discuss this in greater detail in several of the responses below.

2. The Abstract is now an appropriate description of what was done. A minor suggestion is to move the first sentence of the Methods to before the current third sentence (Initially …). This would emphasize that this is a modelling exercise and that exposures or boundaries were not actually determined.

Response:

We agree to your suggestion and have changed the Abstract accordingly.

“Methods: Physical radiation plumes modelled nuclear detonation scenarios of simulated exposures at 22 US locations. Models assumed only location of the epicenter and historical, prevailing wind directions/speeds. The spatial boundaries of graduated radiation exposures were determined by targeted, multistep geostatistical analysis of small population samples...”

3A. One remaining issue in the manuscript body is in the overlap or inappropriate implied equivalence of “individuals” and “locations”. The authors should confirm (by searching for the words) that when talking about measurements or sampling or results that location is applied to physical dosimetry and individual is restricted to biodosimetry. Some of these may just be residual from prior versions but need to be corrected. This would then allow the appropriate inference by the reader that locations are fixed and may accurately reflect physical dose levels at or in proximity to that location. This then leads to a reasonable assumption that interpolation between two locations (by additional sampling) is likely to refine dose boundaries.

Response:

The changes made to the manuscript in our previous revision were to generalize the text so that it could apply to any type of dosimetry method, as long as all measurements in a dosimetry map were obtained using the same method. We state that we are simulating “physical or cytogenetic dosimetry”, as dosimetry data from either method would result in an accurate plume. We have gone through the manuscript again in an attempt to further generalize the text to avoid any confusion about our presentation. Most uses of the term “individuals” have been changed to “samples.” In the Methods section, we define the term “samples” to refer either emissions measured by physical radiation detectors at the time of the nuclear incident or to radiation levels absorbed by biological samples. Since there could be differences between the radiation levels obtained by different methods, we indicate that the dosimetry maps are assumed to be consistently derived by the same method.

The proposed geostatistical method can only be used with dosimetry at known physical locations. This is feasible by design of physical detectors containing GIS devices. In the case of biological samples, the random access memory of cellular telephones carried by individuals should still retain of the last known locations, even in the event of an electromagnetic storm that affects network function. Only biological samples remaining at a fixed location after the nuclear incident would be eligible for geostatistical analysis. The advantages and drawbacks of different dosimetry methods (see next section) do not negate the core claim of this manuscript that the geostatistical method proposed would reduce the required sampling to develop a sufficiently accurate plume for triage purposes.

Table 1 indicates the number of unique samples used to derive our plume. We would like to point out that the number of samples in this table has been decreased significantly. This was due to identical samples being counted as unique if separate densification steps had selected the same location more than once. This was corrected, which reduced the number of samples (from a range of “73 - 417” to “58 – 347”).

3B. The same cannot be said for individuals. A value for a third individual, sampled at a location between the locations where two initial individuals were assessed by biodosimetry, would not a priori be expected to provide an intermediate biodosimetry value. This could be due to heterogeneity in inherent radiosensitivity of the population, movement by the individual, comorbidities, or any of the reasons that the authors allude to. This also leads into the need to be precise as to what measurements are reasonable to utilize for the kriging process and the re-iterative process. That is, one can reasonably postulate additional physical dosimetry sampling over a few days to refine dose boundaries that then can be used to perform biodosimetry on a selected set of individuals. But with the time restraints of biodosimetry it is not practical to consider more than one sampling as contributing to the triaging process. But current wording might suggest otherwise. As an example, at the bottom of page 3, there is the following:
“One approach to alleviating the need to triage all potentially exposed individuals would be to survey a subset of individuals combined with their respective locations by geostatistical analysis. We demonstrate that combining such surveys with the geolocations of these measurements can reduce sampling requirements in population-scale radiation scenarios and would be expected to decrease total radiation exposures of first responders.”
The term “combining” might be interpreted as using the two dosimetry methods reiteratively. Also “surveys” of individuals might be mis-interpreted as multiple surveys (i.e., biodosimetry) in the same general vicinity to aid in the kriging, rather than a single targeted survey of individuals in a location selected by the physical dosimetry kriging.

Response:

Each dosimetry method has advantages and drawbacks but determining the best type of dosimetry method to be used is not (and has never been) the purpose of this study. The objective of the paper is to find a way to accurately map radiation with limited sampling. Our approach could accelerate treatment decisions in a way that has not been previously considered.

There are no optimal dosimetry methods. Applications of biodosimetry may be limited due to the total time required to obtain results, but it does not diminish the value of those results. Complete reliance on physical dosimetry may be concerning, since it does not measure the amount of radiation that has been absorbed and may also be prone to false positives. From the revised Discussion:

“Biodosimetry estimates absorbed exposures, whereas physical dosimeters measure environmental emissions. Physical dosimetry is more rapid and can map changing radiation plume locations dynamically. However, unfiltered radiation emissions are prone to false positive readouts, for example in aerial physical dosimetry counterterrorism surveys (Karam, 2017) due to common environmental sources of radiation. Uncorrected, such data will introduce errors and distort geostatistical-derived plumes. Mitigation may be possible by specifying the locations of radiation detectors by iterative kriging and densification. Nevertheless, biodosimetry at specified locations may provide results that might be useful in assessing treatment eligibility, especially in instances of borderline clinical exposures.”

We specify in the Methods section that sampling of consecutive iterations should use the same approach at each stage of refinement to derive an accurate plume. Our intent is to generalize the text in the manuscript to be agnostic to the dosimetry method used to derive a plume. Changes to the paragraph rectify this as well.

“One approach to alleviating the need to triage all potentially exposed samples would be to survey a subset of samples and their respective locations by geostatistical analysis. This survey may involve location-based physical dosimetry, where high-risk individuals are tagged based on their proximity to nearby detectors. We demonstrate that combining surveys using uniform methodologies with the geolocations of these measurements can reduce sampling requirements in population-scale radiation scenarios. This would also be anticipated to decrease overall radiation exposures to first responders.”

4. Last sentence of Introduction: “kriging using a relatively small number of samples analyzed by physical or biodosimetry methods.” Biodosimetry needs to be removed, but the sentence can be extended to state using kriging of physical dosimetry to aid in selection of individuals for biodosimetry.

Response:

As previously mentioned, we have decided to simplify the text so that the text does not specify the dosimetry method used, as a plume can be derived with any type of dosimetry method as long as it is consistent. We have therefore changed the last sentence of the Introduction to the following:

“The question this paper addresses is whether the radiation plumes derived by HPAC can be reconstructed with iterative kriging using a relatively small number of samples consistently analyzed by the same dosimetry method.”

5. Page 5: “Densification is the geostatistical procedure that targets and localizes an additional small cohort of irradiated individuals (1) to mitigate uncertainty in environmental measurement. These kriging and densification processes are repeated for a limited number of iterations until the coverage area and the radiation level contours of the inferred plume stabilizes (i.e. sampling of additional individuals (2)”. (1) it identifies a location, not any individuals who might be there. (2) Locations will be sampled reiteratively, not individuals.

Response:

. The text has been generalized in the following manner:

- For issue (1): We have changed “irradiated individuals” to “sampling locations”.
- For issue (2): We change “sampling of additional individuals” to “additional sampling”.

This text is now:

“Densification is the geostatistical procedure that targets and localizes an additional small cohort of sampling locations to mitigate uncertainty in environmental measurements. These kriging and densification processes are repeated for a limited number of iterations until the coverage area and the radiation level contours of the inferred plume stabilizes (i.e. additional sampling in the affected area does not significantly alter the geographic coverage of the plume or the estimates of absorbed radiation dose).”

6. Page 7, para 2: “To map predicted radiation levels based on the distribution of dose-estimated patients around the prediction location” It is locations sampled by physical dosimetry that have a distribution – not patients.

Response:

To generalize this sentence, we have changed “patients” to “samples” as this includes physical dose measurements. The sentence now reads as follows:

“To map predicted radiation levels based on the distribution of samples across the location of interest, …”

7. Figure legend 2; last sentence “% of the individuals eligible for cytokine treatment” – locations (weighted for population density) where treatments of individuals might be necessary.

Response:

As suggested, we have edited the sentence to state that we are computing a dose estimate for a location (weighted by population density), rather than for individuals specifically:

“… the converged plumes localized 80.3% and 75% of the locations (weighted for population density) for treatment-eligible radiation exposures in these scenarios. “

8. Page 15; para 2; sentence 1: “subset of irradiated individuals”. Subset of locations - there is no a priori assumption about irradiation of individuals. Sentence 2: unirradiated individuals. Sentence 3: irradiated individuals.

Response:

As previously mentioned, this does not preclude the proposed method from being used to derive radiation plumes based on biologically-determined dose, regardless of assumptions about processing time (see our response to point 11 below). As a compromise, rather than using the word “locations”, we have used the word “samples”, or have modified the sentence to remove the segment which used the word entirely.

9. Page 16; para 2; sentence 1: “incorrect sampling of potentially exposed individuals”. Page 16; para 2; last sentence: “fewer irradiated individuals”.

Response:

Both suggestions pertain to a paragraph discussing dosimetry measurement error. We have modified the first sentence of this paragraph to eliminate the text referring to exposed individuals:

“…it might be expected to lead to improper sampling.”

For the final sentence of the paragraph, we eliminated the word “individuals” and replaced it with “samples”.

10. Page 16, bottom; Page 17 top: The inclusion of variation for biodosimetry, especially for specific methods, is irrelevant (and misleading) – the simulations and the kriging are based solely on physical dosimetry.

Response:

In response to this reviewer’s previous comments, we revised the text to be agnostic to the type of dosimetry method used to obtain the data. As we have indicated (and cited), physical dosimetry is also prone to systematic sources of radiation measurement error. This justifies inclusion of the examples given in the text. We do not agree with the reviewer’s claim that inclusion of variation in radiation measurements is irrelevant and misleading. Indeed, the examples provided illustrate the robustness of the proposed methods to different sources of systematic error. The section on inferred radiation exposures under sub-optimal sampling conditions remains relevant, and we argue, is a strength of our approach. We have therefore included “physical dose estimation error” as an example of possible sources for variation in radiation dose measurements.

“Maximum deviations were designed to represent confidence values in physical and/or biodosimetry methods, including physical dose estimation error (29), dicentric analysis…”

11. Page 19; para 3: This discussion is useful but misses the point. If one can only practically process 160 individuals by biodosimetry (the time frame does not permit going back for a second sample),

Response:

The reviewer appears to assume that First Responses to a nuclear incident regarding sample acquisition and laboratory processing samples will be limited in scope and resources. However, in our previous response to this reviewer, we suggested that field, laboratory and computational resources could be amplified through parallel deployment of multiple dedicated teams and automation. Indeed, we cited a recent, peer-reviewed Concept of Operations article in Radiation Protection Dosimetry that recommended large scale processing and multiple procurement mechanisms. We suggest that the reviewer consider such alternatives, which could overcome the capacity and time limitations that s/he envisions in a large scale nuclear incident.

11. (continued) then one must focus on those most likely to be shown to irradiated (> 2 Gy). And one must have this subset within a few days at most (allowing how many rounds of physical dosimetry for kriging?). Beyond that, one is too late to initiate cytokine therapy with any expectation of success.

Response:

Our paper does not model how long dosimetry testing and measurement will require or what type of testing (physical or biological) will be performed. The reviewer’s assumptions about what would be feasible to accomplish within a 3 day time frame seem to be predicated on a single field team sampling exposed individuals or placing radiation detectors followed by sequential geostatistical analysis. With high performance computing and parallel processing, it should be possible to model different data sources at the same time, and then combine these into more robust geostatistical models. It is really beyond the scope of this first publication on the subject (nor do we have the resources to perform at this time) large scale modeling of multi-source data processing and geostatistical analysis. Nevertheless, we stand by the main conclusions of the paper, that strategic sampling guided by geostatistical methods of a relatively small fraction of irradiated “samples” can be used to compute exposures for much larger populations.

11. (continued) Also unaddressed is whether the proposed biodosimetry techniques are effective for assessing cumulative chronic doses (over a few weeks) – or whether they have all been developed using a specific (24 hr?) time point of sampling after a single acute radiation exposure. If that is the case, then even 3-4 days for physical dosimetry kriging may not be beneficial

Response:

We state that biodosimetry does not address dynamic changes in radiation levels which have cumulative effects. Physical dosimetry can provide dynamic measurements of emitted radiation, but does not measure how much radiation is absorbed. In any case, distinctions about intermediate or long term exposures are not relevant in a triage situation where the existing testing capacity may not be sufficient to process every individual or sample that would be desirable to create a dense dosimetry plume map.

12. A final major issue that is subject to misinterpretation by an audience that is not familiar with the radiation countermeasures/response field is contained in the last sentence of Figure legend 1. ”The individuals residing in this part are eligible to be treated by cytokine therapies.” This implies that treatment (with potentially deleterious effects) would be administered to individuals on the basis of either a physical dosimetry measurement map that includes their location or even a biodosimetry assessment of a neighbor – but not themselves. This is most likely to be the case. Or if the authors, believe that it would be, they should provide a citation for the strategy.

Response:

We concur that the derived map would not be used – by itself - as the sole basis of managing treatment. The described method is designed to triage and identify individuals who may benefit from treatment in situations, based on limited sampling of irradiated individuals in the affected geographic regions. The Introduction of the paper describes multiple clinical criteria that contribute to the diagnosis of Acute Radiation Syndrome, and nothing in the paper disputes any of these criteria. Besides symptoms, laboratory testing would contribute valuable confirmation of these symptoms (especially if they were mild or non-specific due to presence of other confounding diagnoses) may treatment, which may include cytokine therapies.

To avoid any confusion or misinterpretation, we have decided to delete this sentence entirely from the Figure 1 legend, as it is unrelated to the content of Figure 1. Furthermore, the significance of the >2Gy threshold has been stated elsewhere in the manuscript.

13. In all, the authors present what could be a valuable aide in directing triage and treatment of selected individuals in the aftermath of a nuclear scenario. But it must be presented reasonably and without the potential to confuse and mislead a readership that will be predominately be unfamiliar with any radiation dosimetry methods or with the strategies likely to be employed in such a situation.

Response

We modified the paper according to the reviewer’s recommendations to avoid endorsement of any particular dosimetry method. We believe that those deciding what strategy to employ after a nuclear incident should not be bound by previous dogma about which types of dosimetry to perform and when. Further research in this area could address optimization of geostatistical analysis when multiple dosimetry data types are collected.

However, the purpose of this study was to introduce this approach and demonstrate using simulations that it could provide a means of inferring radiation across the landscape given a limited set of assumptions (epicentre, wind vector, and weather), and fewer radiation measurements. For us to provide a complete TR7-level solution at this stage would be beyond the scope of this paper.

**Reviewer #2:**

The authors use geospatial modeling in an attempt to decrease the need for radiation dosimetry in the setting of a large scale disaster. They modeled 30 scenarios, including 22 urban/high-density and 2 rural/low-density scenarios under various weather conditions. Multiple (3-10) rounds of sampling and kriging were required for the dosimetry maps to converge, requiring between 73 and 417 samples for different scenarios. On average, 70±10% of locations where populations are expected to receive an exposure ≥2Gy were identified. They conclude that geostatistical mapping limits the number of individuals requiring dose assessment, the time required, and radiation exposure to first responders. Geostatistical analysis will expedite triaging of acute radiation exposure in population-scale nuclear events.

This manuscript is a highly mathematical approach to a major problem in a mass casualty event concerning radiation exposure. The major hurdle with this reviewer is that this approach does not really solve the problem. Based on their data from table 1, the range is so variable that this reviewer does not see the value of this approach. For example, the accuracy is as low as in the 20% range making this approach inferior to a coin toss. The best case scenario is in the low 80% range. I am not clear that this provides much more information since it’s not clear what the responders would approach the other 20% that did receive a higher dose.

Response:

We reject the notion our “mathematical approach“ should be expected to completely identify every single individual with clinically relevant radiation exposure levels. We anticipate that it will be primarily applied in triage situations soon after a nuclear incident, with the primary focus of providing guidance for an orderly and efficient response in the early stages of in a large scale nuclear incident.

The reviewer misstated the performance of our best case scenario which was actually 90.7% overlap of the Burlington VT HPAC plume at the 2Gy plume contour. The current version of the manuscript now demonstrates that the accuracy of overlap between plumes –where it counts- at higher radiation levels (3 Gy) is higher for most of the scenarios that we present. Nearly all of the contours are contiguous and non-intersecting, that is 3 Gy exposures which are miscalled are still interpreted as >2 Gy. Although incorrectly determined high level exposures are slightly underestimated, such individuals will still be brought to clinical attention.

To the best of our knowledge, this is the first report of this approach for triaging radiation exposed individuals for sample measurement. While it may not be perfect, we present a novel, useful and scientifically valid solution to a longstanding problem. Perfection is likely unachievable. Indeed, as we describe, physical radiation measurements and biodosimetry testing also contribute systematic error, and therefore even the most comprehensive saturation testing protocols will be susceptible to such errors.

We have revised and ensured that this paper meets the criteria for publication in PLoS ONE. Specifically, the simulations we performed have been conducted rigorously, with appropriate replication, and we have presented a wide variety of scenarios to explore the strengths and weaknesses of the proposed approach. The data presented in the manuscript support the conclusions drawn and are not overstated.  Methods and data have been deposited in publicly available resources. In fairness, we ask the Reviewer to consider the publication criteria that the journal has established in assessing our contribution.

We have been honest and transparent about sources and impacts of error in radiation measurements and limitations of the approach for computing accurate low level radiation exposures. For example, the census data that is publicly available is not as granular as the data that the US Census Bureau actually collects. Thus, when we compute the number of samples that would be impacted by a radiation plume, this requires us to compute the population density for the minimal reported geographic region, which is usually a county subdivision. This averages impact over a geographic area and affects the accuracy of our estimates. Small overlapping segments of subdivisions are particularly prone to these types of errors. Also, we show that the reported accuracy does depend on the fraction of the total population that is initially sampled, and that sparsely populated regions are more prone to lower levels of accuracy (see below).

While the majority of scenarios gave results consistent with the HPAC plume, there were 2 exceptions. We have performed additional work that provides an explanation of the particular Columbia SC and Columbus OH anomalous replicates which did not reproduce the HPAC plume (Results, 3^rd^ paragraph, Discussion 2^nd^ paragraph). Initial sampling for these replicates exhibited sparse coverage over large regions of the HPAC plume. Densification steps did not select locations for further sampling. Scenarios with low population density appear to be susceptible to this problem. We demonstrate that it can be mitigated by either increasing sampling density, or by strategically selecting sampling locations within this region. From the Discussion:

“In a real-world scenario, secondary sampling locations assigned by densification would be supervised, which would direct the software towards derivation of a complete and accurate plume. Indeed, we found that manually adding two new sample locations to the unrepresented region corrected the poor results obtained for the Columbia SC scenario replicate after two additional cycles of kriging and densification. “

Lastly, a major difficulty is partial body shielding which would make this approach even more difficult to interpreted.

Response:

Reviewer #2 had previously mentioned shielding during the first round of revisions:

“The major concern is that the radiation doses in a large city will be inhomogeneous due to partial shielding. How does this algorithm account for such events?”

To which we responded:

The Discussion describes the limitations of our method. Here, we discuss how shielding (or “shadowing”) by infrastructure is not accounted for, and how these concessions may limit the overall accuracy of our method.

Results (Paragraph 3, page 18): “Finally, neither the HPAC version available for this study, nor our geostatistical models account for shielding by infrastructure, such as shadowing, which computes the degree to which radiation is prevented from reaching certain locations by the urban environment (35). While these factors will impact the predicted accuracies of derived dosimetry maps, these effects will also have to be accounted for in ground truth models, such as HPAC, before they could be addressed in geostatistical interpolation.”

During the second round of revisions, it seemed that this response had satisfied the reviewer:

“Reviewer #2: all concerns addressed - no further comments”

In the future, obstacles that occlude diffusion of radioactive particles could be factored into the derived plume. Samples from city neighborhoods with densely arrayed, tall building infrastructure may be partially shielded, resulting in lower actual exposures to ionizing radiation. However, we did not and still do not have access to these calculations to test this possibility, since these algorithms and software, to the best of our knowledge, are not publicly available.
